# Factors associated with academic-medicine faculty attrition before promotion to senior rank: A national-cohort study

**DOI:** 10.1371/journal.pone.0355830

**Published:** 2026-09-02

**Authors:** Maria Pérez, William E. Freeman, Yaheng Lu, Donna B. Jeffe

**Affiliations:** Washington University School of Medicine, St. Louis, Missouri, United States of America; University of South Dakota Sanford School of Medicine, UNITED STATES OF AMERICA

## Abstract

**Background:**

Women and certain groups remain underrepresented among academic-medicine faculty at senior ranks (associate professor or professor). Variables associated with attrition before promotion to senior rank remain understudied.

**Methods:**

The Association of American Medical Colleges provided data for a national cohort of 129,860 U.S. MD-granting medical-school matriculants in academic years 1993–1994 through 2000–2001; the authors included graduates from 1997 through 2012, first appointed to full-time instructor or assistant professor between 2000 and 2020. Independent predictors of attrition before reaching senior rank (vs. retention, with or without promotion to senior rank) were identified using multivariable logistic regression analysis.

**Results:**

Of 35,902 graduates appointed to instructor or assistant professor, 34,478 (96.0%) had complete data for analysis. Faculty were more likely to leave academic medicine before promotion to senior rank if they were female (vs. male), Asian, and underrepresented groups in medicine (each vs. White), had any (vs. no) debt, reported non-academic (vs. full-time faculty) career plans at graduation, were first appointed as instructor (vs. assistant professor), at institutions where tenure was unavailable (vs. not on tenure track), and in a Family Medicine or Psychiatry (vs. Medicine) department. Faculty were less likely to leave academic medicine before promotion to senior rank if they had at least one year (vs. no) research during residency, were first appointed at research-intensive (vs. non-research-intensive) institutions, in a Pediatrics or Surgery (vs. Medicine) department, and had received any (vs. no) research or other federal grants.

**Conclusion:**

This national-cohort study contributes new knowledge to the literature about variables independently associated with academic-medicine attrition before promotion to senior rank, many of which are amenable to intervention. Programs that can foster students’ and trainees’ interest in academic-medicine careers, reduce debt, and promote grant-writing skills might reduce attrition before reaching senior rank and increase academic-medicine workforce diversity.

## Introduction

Physicians in academic medicine have opportunities to deliver patient care, educate and mentor future physicians and potential faculty, and engage in ground-breaking research. However, racial and ethnic disparities in the promotion and retention of academic-medicine faculty in the United States (U.S.) have been reported [1–3]. Lower probabilities of promotion and higher probabilities of attrition have been reported among groups historically underrepresented in medicine (URiM) [4], leading to a lack of URiM faculty at senior rank (associate or full professor) across medical specialties [5]. Racial, ethnic, and sex disparities in representation among academic-medicine physician faculty have been observed across faculty ranks, medical specialties, and basic science and clinical departments [5–12], exacerbating the lower representation of URiM and female faculty in academic medicine at senior rank. For example, male and non-URiM emergency medicine physicians have been shown to be more likely to hold academic ranks as professor or associate professor compared with female and URiM faculty [11]. Male urologists were reported to have been promoted from assistant to the associate professor rank at least a year earlier, on average, than female urologists [9]; however, this study did not account for reasons, such as time taken as leave for “nonwork purposes,” which might explain the disparity in time to promotion between male and female physicians in their sample. Indeed, male faculty have been reported to be promoted more often and more quickly to associate or full professor than female faculty across basic science and clinical departments [8].

Promotion to senior rank not only recognizes physicians’ clinical achievements, leadership, and scholarly contributions to their field, it is important to the tripartite mission of academic medicine [13,14], as senior faculty serve as essential institutional resources as educators and mentors to medical students, residents, and fellows [15]. Many studies investigating promotion to senior rank, however, were cross-sectional [6,7,9–11]. Longitudinal studies examining sex, racial, and ethnic disparities in promotion also have been limited by comparing promotion rates with expected rates based on their representation in the U.S. population using Census data [5] or based on a group’s representation among graduating students in their class [8]. Other studies were limited by not accounting for variables known or expected to be associated with faculty promotion, including faculty track or tenure status [3], or by excluding certain groups of academic physicians altogether, such as those first appointed as instructor [5,8], who accounted for 8.4% of all faculty appointments in clinical and basic science departments in 2023 [16].

Furthermore, there is a paucity of national-cohort studies of the academic-medicine workforce examining factors associated with attrition specifically, which could promote retention of underrepresented groups in academic medicine [4]. Notably, a national study, using data from the Centers for Medicare & Medicaid Services for all physicians in teaching hospitals who billed Medicare from 2014 to 2019, reported only on gender differences in attrition rates, with women leaving academic medicine at higher rates than men across all career stages [17]. The authors of that study defined attrition as not billing Medicare for more than one year during the study period, excluding retirees; however, the extent to which variables that could be changed to mitigate attrition was not examined [17]. Thus, we conducted a national-cohort study of all matriculants in U.S. medical schools with long-term follow-up to identify individual and institutional factors that are amenable to intervention and that were hypothesized, based on the literature, to be associated with physicians’ attrition from academic medicine before promotion to senior rank.

## Methods

### Design

Association of American Medical Colleges (AAMC) staff provided de-identified and individually linked data from various sources in 2009 for all 129,860 matriculants in U.S. Liaison Committee on Medical Education (LCME)-accredited medical schools during academic years (AYs) 1993–1994 through 2000–2001. For analysis, we included graduates in calendar years between 1997 and 2012, who were appointed to full-time instructor or assistant professor faculty positions at LCME-accredited medical schools between 2000 and 2020. We obtained follow-up data from the AAMC Faculty Roster data on April 3, 2020, which allowed for up to 19 years of follow-up *after initial appointment*. This study of de-identified data was reviewed by the Institutional Review Board (IRB) at Washington University School of Medicine and was approved as non-human-subjects research (IRB # 201808151). We followed the Strengthening the Reporting of Observational Studies in Epidemiology (STROBE) reporting guidelines [18].

### Measures

From the American Medical College Application Service® [19], we received data for self-identified sex (male or female) and race/ethnicity (categorized as White, Asian, or URiM [including Black/African American, Hispanic/Latino, American Indian/Native Alaskan, Native Hawaiian/Other Pacific Islander]). At the time of data collection, only one option for race could be selected. We also obtained data from the AAMC for degree program at graduation (MD, including BA/BS-MD; MD-PhD; or MD-other-advanced degrees).

From the AAMC Matriculating Student Questionnaire [20], voluntarily completed at matriculation, we received data for parents’ education (first-generation college graduate [neither parent completed 4-year BA/BS degree], continuing-generation college graduate [at least one parent completed BA/BS degree with or without further graduate or professional education], or both parents’ education was unknown). MSQ response rates for this cohort were 93.5% (Matthew D, AAMC Data Operations and Services, email communication, August 30, 2022).

From the AAMC Graduation Questionnaire [21], voluntarily completed by graduating medical students, we received data for career plans at graduation (full-time university faculty in basic or clinical sciences; non-academic clinical practice/other/non-university research scientist/undecided; and missing), and total debt at graduation (no debt, $1-$99,999, ≥ $100,000, or missing). GQ response rates for this cohort averaged 74.0% (Matthew D, AAMC Data Operations and Services, email communication, August 30, 2022).

From the AAMC’s Graduate Medical Education (GME) Track database [22], we received data about whether a resident participated in at least one year of dedicated research during GME. Accreditation Council for Graduate Medical Education–accredited residency program directors and institutional officials voluntarily complete the National GME Census, with annual response rates averaging 92.6% (Roskovensky L, AAMC Data Operations and Services, email communication, September 2023).

The AAMC acquired information about federal grant awards received from the National Institutes of Health (NIH) Information for Management Planning Analysis and Coordination (IMPAC II) database, linked these data for individuals in our database, and provided the de-identified data to us in August 2014. We created a 3-category variable for analysis of grants awarded to an individual prior to promotion to senior rank or to attrition from academic medicine (never received any grants, received R01 and/or research project grants (RPGs), or received only non-RPG grants [23], including pre- or post-doctoral (e.g., F, K, T, U, or other) grants.

The AAMC also provided data about whether a student attended a research-intensive medical school (yes or no) and whether a faculty member’s appointment was at a research-intensive institution (yes or no). These institutional variables were determined by each school’s annual ranking in the Top 40 for direct research expenditures from federal grants and contracts using data from the LCME Part I-A Annual Financial Questionnaire, which the AAMC administers [24].

The AAMC Faculty Roster data [25] included start and end dates of first and subsequent full-time faculty appointments, initial rank (i.e., instructor or assistant professor) and subsequent faculty ranks, and track at first appointment (nontenure-eligible track, tenure-eligible track without tenure, tenure not available at the institution, or missing). Faculty first appointed with tenure were excluded. The AAMC also provided data for department at first appointment; we created a 9-category variable for analysis (Internal Medicine, Family Medicine, Pediatrics, Obstetrics and Gynecology [OB/GYN], Surgery, Psychiatry, Neurology, Facilities-based specialties [i.e., Anesthesiology, Emergency Medicine, Pathology, Radiology], and all other clinical and nonclinical departments combined). We expected the number of months of follow-up after first appointment to be associated with attrition, as faculty more recently appointed may be less likely to leave academic medicine. We used faculty appointment start and end dates to compute the number of months of follow-up after first appointment until promotion to senior rank, attrition from academic medicine without promotion to senior rank, or date of censorship (February 29, 2020, if retained in academic medicine without promotion to senior rank). We created a dichotomous outcome variable comparing attrition from academic medicine, defined as leaving a full-time faculty position prior to promotion to senior rank, versus retention in a full-time academic-medicine faculty position, with or without promotion to senior rank.

### Data analysis

We used chi-square tests to measure associations between our binary outcome (attrition vs. retention, with or without promotion to senior rank) and each categorical variable of interest, reporting frequencies (%). Analysis of variance was used to test the difference in mean (SD) number of months of follow-up after initial appointment by our outcome. A multivariable logistic regression model, controlling for number of months of follow-up after first appointment, was run to identify independent predictors of attrition from academic medicine (between-subjects variables) before promotion to senior rank (vs. faculty retention). We report adjusted odds ratios (aORs) and 95% confidence intervals (CIs); two-tailed p-values < 0.05 were considered statistically significant. Analyses were performed using IBM SPSS Statistics, version 29.0.2.0 (IBM Corp., Armonk, NY).

## Results

**Fig 1:**
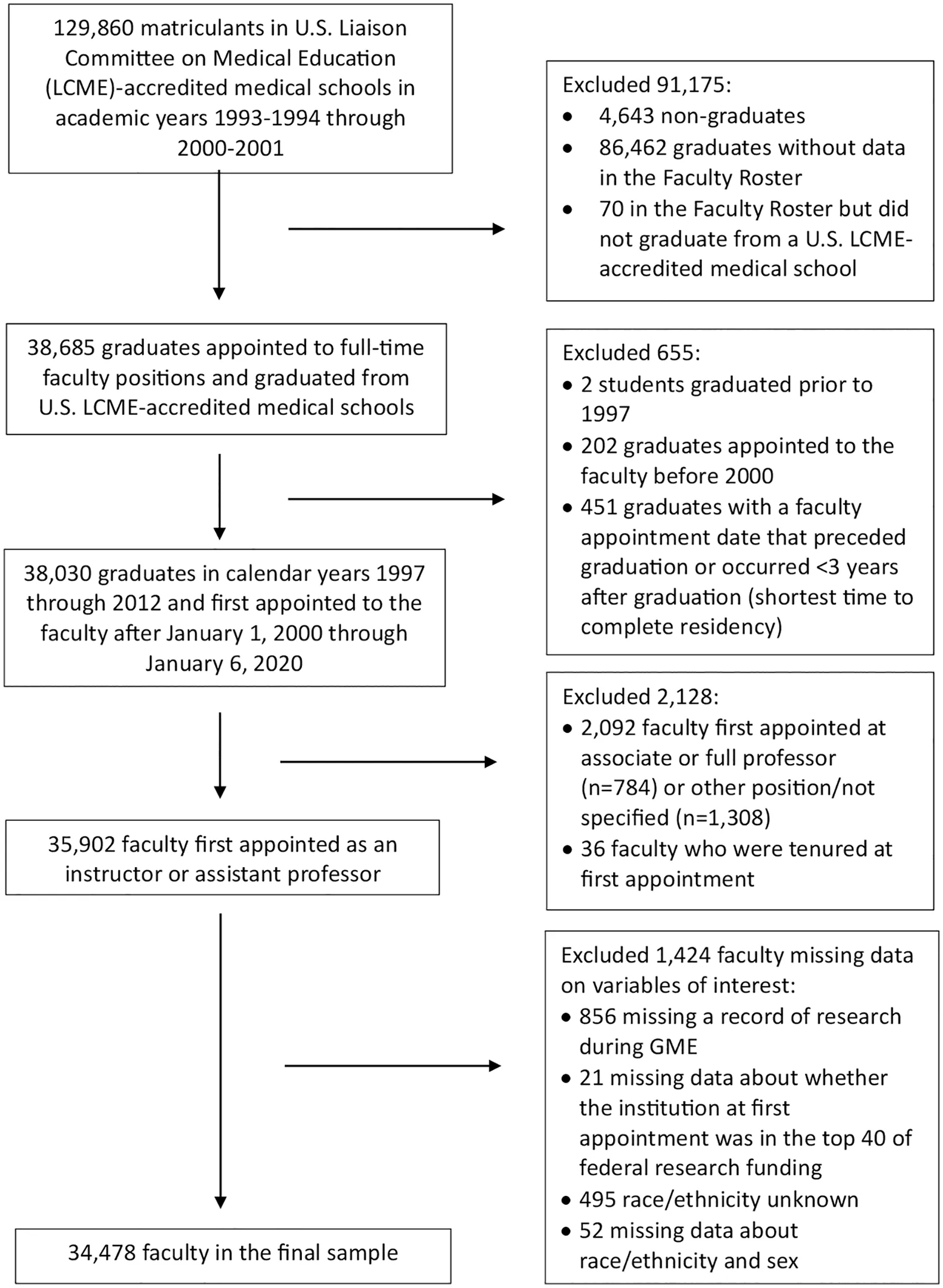
Flow Diagram for Cohort Selection for Eligibility and Inclusion in Analysis.

Of 129,860 matriculants in this national cohort, 4,643 students did not graduate and were excluded. Among the 125,217 graduates, 38,685 (30.9%) had a record of full-time faculty appointment in the Faculty Roster. Of 38,030 graduates in calendar years 1997–2012 who were appointed to the faculty between 2000 and 2020, 35,902 (94.4%) were first appointed to instructor or assistant professor and eligible for inclusion. Fig 1 illustrates the derivation of our final sample (N = 34,478) with complete data for analysis, which includes 31,968 MD, 1,934 MD-PhD, and 576 MD-other-advanced-degree graduates.

In this sample, significantly greater proportions of female (vs. male) and of Asian and URiM (each vs. White) faculty left academic medicine before being promoted to senior rank. Potential covariates of interest (Table 1) were significantly associated with attrition from academic medicine before promotion to senior rank and were included in the regression model.

**Table 1:** Tests of association between attrition (leaving a full-time faculty position) from academic medicine before attaining senior rank (vs. retention with or without promotion) and other variables among U.S. LCME-accredited medical-school graduates in calendar years 1997 through 2012. who were first appointed to the faculty as an instructor or an assistant professor in 2000 through date of censorship, April 3, 2020 (N = 34,478).

	N (%)	Attrition n (%)	Retention n (%)	p-value^a^
**Gender**				< 0.001
Male	18,932 (54.9)	7,354 (38.8)	11,578 (61.2)	
Female	15,546 (45.1)	6,788 (43.7)	8,758 (56.3)	
**Race/ethnicity**				< 0.001
White	23,119 (67.1)	9,169 (39.7)	13,950 (60.3)	
Asian	7,002 (20.3)	2,891 (41.3)	4,111 (58.7)	
URiM	4,357 (12.6)	2,082 (47.8)	2,275 (52.2)	
**Parent education**				< 0.001
Continuing-generation college graduate	26,931 (78.1)	10,766 (40.0)	16,165 (60.0)	
First-generation college graduate	5,120 (14.9)	2,371 (46.3)	2,749 (53.7)	
Both parents’ education unknown	2,427 (7.0)	1,005 (41.4)	1,422 (58.6)	
**Attended research-intensive medical school**				< 0.001
No	20,173 (58.5)	9,050 (44.9)	11,123 (55.1)	
Yes	14,305 (41.5)	5,092 (35.6)	9,213 (64.4)	
**Career plans at graduation**				< 0.001
Full-time faculty in basic or clinical sciences	12,857 (37.3)	4,353 (33.9)	8,504 (66.1)	
Non-academic clinical practice, other, non-university research scientist, undecided	15,559 (45.1)	7,288 (46.8)	8,271 (53.2)	
Missing	6,062 (17.6)	2,501 (41.3)	3,561 (58.7)	
**Total debt at graduation**				< 0.001
No debt	5,304 (15.4)	1,935 (36.5)	3,369 (63.5)	
$1–$99,999	12,013 (34.8)	4,889 (40.7)	7,124 (59.3)	
≥$100,000	10,908 (31.6)	4,770 (43.7)	6,138 (56.3)	
Missing	6,253 (18.1)	2,548 (40.7)	3,705 (59.3)	
**Record of ≥1 year of GME research**				< 0.001
No	27,276 (79.1)	12,008 (44.0)	15,268 (56.0)	
Yes	7,202 (20.9)	2,134 (29.6)	5,068 (70.4)	
**Rank at first appointment**				< 0.001
Assistant professor	22,966 (66.6)	8,203 (35.7)	14,763 (64.3)	
Instructor	11,512 (33.4)	5,939 (51.6)	5,573 (48.4)	
**Track at first appointment**				< 0.001
Not on tenure-eligible track	23,733 (68.8)	9,424 (39.7)	14,309 (60.3)	
On tenure-eligible track	5,487 (15.9)	2,017 (36.8)	3,470 (63.2)	
Tenure not available	2,183 (6.3)	960 (44.0)	1,223 (56.0)	
Missing	3,075 (8.9)	1,741 (56.6)	1,334 (43.4)	
**Grants received before promotion to senior rank or attrition before senior rank**				< 0.001
Never had any grant	31,935 (92.6)	13,779 (43.1)	18,156 (56.9)	
Only non-RPGs	1,927 (5.6)	301 (15.6)	1,626 (84.4)	
R01 and/or other RPGs, with or without non-RPGs	616 (1.8)	62 (10.1)	554 (89.9)	
**Department at first appointment**				< 0.001
Internal Medicine	9,235 (26.8)	3,689 (39.9)	5,546 (60.1)	
Facilities-based (Anesthesiology, Emergency Medicine, Pathology, Radiology)	6,882 (20.0)	3,199 (46.5)	3,683 (53.5)	
Surgery	6,189 (18.0)	2,211 (35.7)	3,978 (64.3)	
Pediatrics	5,097 (14.8)	1,796 (35.2)	3,301 (64.8)	
OB/GYN	1,820 (5.3)	810 (44.5)	1,010 (55.5)	
Psychiatry	1,629 (5.1)	888 (50.2)	880 (49.8)	
Family Medicine	1,269 (3.7)	682 (53.7)	587 (46.3)	
All other clinical and nonclinical departments	1,254 (3.6)	548 (43.7)	706 (56.3)	
Neurology	964 (2.8)	319 (33.1)	645 (66.9)	
**First faculty appointment at a research-intensive medical school**				< 0.001
No	11,125 (32.3)	5,128 (46.1)	5,997 (53.9)	
Yes	23,353 (67.7)	9,014 (38.6)	14,339 (61.4)	
	Mean (SD)	Mean (SD)	Mean (SD)	p-value^b^
**Months of follow-up since faculty appointment**	80.6 (46.8)	52.9 (39.2)	99.9 (41.7)	< 0.001

Abbreviations: LCME, Liaison Committee on Medical Education; URiM, Underrepresented in Medicine (Black/African American, Hispanic/Latino, American Indian/Native Alaskan, Native Hawaiian/Other Pacific Islander); GME, graduate medical education; RPG, research project grant; OB/GYN, Obstetrics and Gynecology

^a^p-values from chi-square tests of significance.

^b^p-value using analysis of variance.

Multivariable logistic regression model results of variables independently associated with attrition from academic medicine before senior rank are shown in Table 2. We observed a greater likelihood of attrition before promotion to senior rank among female (vs. male), URiM and Asian (each vs. White), first-generation (vs. continuing-generation) college graduates, among GQ respondents who reported non-academic clinical practice/other/non-university research scientist/undecided (vs. full-time faculty) career plans, and having any level of debt at graduation (vs. no debt). A greater likelihood of attrition was also observed among faculty at institutions where tenure was not available (vs. not on the tenure track) as well as faculty first appointed as instructors (vs. assistant professor) and in each of Family Medicine and Psychiatry departments (each vs. Medicine).

**Table 2:** Multivariable logistic regression analysis to identify variables independently associated with attrition from academic medicine before attaining senior rank (vs. retention with or without promotion) among U.S. LCME-accredited medical-school graduates in calendar years 1997 through 2012, who were first appointed to the faculty as instructor or assistant professor in 2000 through date of censorship, April 3, 2020 (N = 34,478).

	Attrition (vs. retention) aOR (95% CI)^a^	p-value
**Gender**		
Male	1.00 (reference)	
Female	1.28 (1.21-1.36)	< 0.001
**Race/ethnicity**		
White	1.00 (reference)	
Asian	1.13 (1.05-1.20)	< 0.001
URiM	1.31 (1.21-1.43)	< 0.001
**Parent education**		
Continuing-generation college graduate	1.00 (reference)	
First-generation college graduate	1.19 (1.10-1.28)	< 0.001
Both parents’ education unknown	0.97 (0.87-1.07)	0.50
**Attended research-intensive medical school**		
No	1.00 (reference)	
Yes	0.76 (0.72-0.81)	<0.001
**Career plans at graduation**		
Full-time faculty in basic or clinical sciences	1.00 (reference)	
Non-academic clinical practice, other, non-university research scientist), undecided	1.33 (1.26-1.42)	< 0.001
Missing	1.63 (1.33-2.00)	< 0.001
**Total debt at graduation**		
No debt	1.00 (reference)	
$1–$99,999	1.16 (1.06-1.25)	< 0.001
≥$100,000	1.21 (1.11-1.31)	< 0.001
Missing	0.74 (0.60-0.92)	0.006
**Record of ≥1 year of GME research**		
No	1.00 (reference)	
Yes	0.69 (0.64-0.74)	< 0.001
**Rank at first appointment**		
Assistant professor	1.00 (reference)	
Instructor	3.07 (2.89-3.27)	< 0.001
**Faculty track at first appointment**		
Not on tenure-eligible track	1.00 (reference)	
On tenure-eligible track	1.05 (0.98-1.13)	0.19
Tenure not available	1.28 (1.15-1.43)	< 0.001
Missing	1.54 (1.40-1.70)	< 0.001
**Grants received before promotion to senior rank or attrition before senior rank**		
Never had any grant	1.00 (reference)	
Only non-RPGs	0.37 (0.32-0.42)	< 0.001
R01 and/or other RPGs, with or without non-RPGs	0.24 (0.18-0.32)	< 0.001
**Department at first appointment**		
Internal Medicine	1.00 (reference)	
Facilities-based (Anesthesia, Emergency Medicine, Pathology, Radiology)	0.96 (0.89-1.04)	0.33
Surgery	0.63 (0.58-0.68)	< 0.001
Pediatrics	0.82 (0.75-0.90)	< 0.001
OB/GYN	0.88 (0.77-0.995)	0.04
Psychiatry	1.39 (1.23-1.58)	< 0.001
Family Medicine	1.31 (1.13-1.51)	< 0.001
All other clinical and nonclinical departments	0.81 (0.70-0.94)	0.004
Neurology	0.70 (0.59-0.83)	< 0.001
**First faculty appointment at a research-intensive medical school**		
No	1.00 (reference)	
Yes	0.91 (0.85-0.96)	0.001
**Months of follow-up since faculty appointment**	0.97 (0.969-0.971)	< 0.001

Abbreviations: LCME, Liaison Committee on Medical Education; aOR, Adjusted odds ratio; CI, Confidence interval; URiM, Underrepresented in Medicine (Black/African American, Hispanic/Latino, American Indian/Native Alaskan, Native Hawaiian/Other Pacific Islander); GME, graduate medical education; RPG, research project grant; OB/GYN, Obstetrics and Gynecology

A lower likelihood of attrition was observed among faculty who attended a research-intensive (vs. non-research-intensive) medical school, participated (vs. did not participate) in ≥1 year of GME research, were appointed to the faculty at a research-intensive (vs. non-research-intensive) institution, received an R01 and/or other RPGs or received only non-RPGs (each vs. no grants), and were appointed in a Pediatrics, OB/GYN, Surgery, Neurology, or all other clinical and nonclinical departments combined (each vs. Medicine).

## Discussion

Our study adds to the literature in novel ways as we examined factors independently associated with physician-faculty’s attrition from academic medicine before reaching senior rank using an extensive, unique, national-cohort database of U.S. medical-school matriculants with 8–23 years follow-up *after graduation*. To our knowledge, no studies like this have reported on factors associated with attrition using a large, national-cohort database of medical-school matriculants. Several variables we identified, including total debt at graduation, having research and authorship experiences during medical school and GME that can promote interest in and prepare trainees for academic careers, and receiving medical school training at or being first appointed to the faculty at a research-intensive institution, are discussed in the context of the literature. These variables, in particular, are amenable to intervention, which may help increase the diversity of the physician faculty in academic medicine.

### Demographics

In this study, we observed a greater likelihood of attrition before reaching senior rank among faculty who were female, Asian, URiM, and first-generation college graduates. Although increasing numbers of women have received faculty appointments over the years, there remain substantial sex disparities in career-advancement opportunities and salary compensation [26–28], which may lead women to leave academic medicine. Others have also reported Asian and URiM faculty are at greater risk of attrition compared with White faculty [4,12], and reasons for attrition may differ between Asian and URiM groups. Asian American medical students have reported exclusion from campus diversity efforts [29], and such experiences may persist as junior faculty. Therefore, supportive resources designed to promote retention of Asian faculty may be warranted [30], with some suggesting tailoring interventions, like mentoring, for different groups of faculty whose needs might vary [31]. Mentoring skills can be taught and can foster faculty retention and promotion [32,33]. Mentorship is perhaps especially important for groups known to be at risk for attrition. Training a diverse cadre of mentors, tailoring mentoring programs to meet specific faculty needs, and providing resources to promote retention were each reported to support the success of Asian and URiM faculty in academic medicine [34]. Furthermore, first-generation college graduates, many of whom were also from URiM groups [35], have reported a lack of institutional support during medical school, including lack of career advice and educational and psychological support [36], which also may be experienced after faculty appointment. Several publications of successful, national, research-mentoring programs, such as the NIH National Heart, Lung, and Blood Institute-funded PRIDE initiative, demonstrated promising results. These programs helped promote success in receipt of research grant awards, publications, faculty promotion and retention of a diverse cadre of faculty in academic medicine as well as reporting satisfaction with mentoring and increases in research self-efficacy [37–40]. Rigorous evaluations of faculty-mentoring programs designed to enhance faculty promotion and retention are needed to highlight institutions’ return on investment to justify ongoing support for such programs [41]. Faculty-mentoring programs that train faculty to become mentors also require evaluation [42].

### Career plans and total debt at graduation

Faculty who planned any non-academic career upon medical-school graduation were more likely to leave academic medicine before reaching senior rank, similar to an earlier study of initial promotion and attrition with shorter follow-up [4]. Experiences during medical school can contribute to students’ consideration of academic-medicine careers [43–46]. For example, medical students’ engagement in research/authorship was associated with faculty receipt of career-development and other grant awards [47,48], and graduates who attended research-intensive (vs. non-research-intensive) medical schools were more likely to be appointed to full-time faculty positions in academic medicine [49,50]. Similarly, MD-PhD graduates who attended medical schools with federal Medical Scientist Training Program (MSTP) funding (vs. non-MSTP-funded) were more likely to be appointed to full-time faculty positions in academic medicine [51]. Thus, interventions during medical school that encourage and prepare students to pursue academic careers not only increase the likelihood of faculty appointment [50], but they also can have lasting impact thereby promoting retention.

That faculty who reported debt at graduation were more likely to leave academic medicine before reaching senior rank is not surprising, given the notable salary differences between academic physicians and physicians in private practice [52]. Medical-education-related debt is burdensome and has been associated with a lower likelihood of choosing an academic-medicine career among family medicine trainees [53]. Even before GME, the effect of debt was observed among medical students’ plans at graduation to pursue academic-medicine careers, with lower levels of debt < $150,000 showing lower odds of diminished interest and greater odds of sustained interest in pursuing academic-medicine career [43]. URiM residents across all specialties were more likely to have debt, in general, than their White and Asian counterparts [54]. Participating in loan-repayment programs could help reduce debt burden, although physician applications for loan repayment programs had declined by more than 30% between 2011–2020 [55]. Multiple interventions, such as educational scholarships, financial guidance/planning for trainees and early-career faculty, and loan-forgiveness programs, may be necessary to reduce debt-related stress and perhaps promote initial interest in an academic-medicine career and retention of physicians on the faculty and in the biomedical-research workforce [4,54].

### Research-intensive institutions

Attending a research-intensive medical school and having a first faculty appointment at a research-intensive institution were independently associated with a lower likelihood of attrition before reaching senior rank. Attending and being employed at research-intensive medical schools may be beneficial to junior faculty given the broad availability of resources, including research mentoring and faculty-development programs, which are intended to promote retention in biomedical-research and academic careers. Many research-intensive medical schools, especially those with MSTP funding, have clear mentoring program objectives and processes to support MD/PhD students in order to achieve educational program success [56,57], and graduates with dual MD/PhD degrees have been observed to be more likely to hold full-time faculty appointments [58]. All medical students, trainees, and faculty at schools with an MSTP-funded MD/PhD training program also likely benefit from the strong institutional support for research at research-intensive medical schools.

### Research experiences and grant funding

A recent review about surgeon-scientist trainees’ experiences noted that GME-research years often support trainees’ professional development via education and mentoring in grant writing, applying for funding, structured research experiences, and career mentoring [59]. Experiences that trainees obtain during their research year(s) likely set them ahead of their peers in navigating hurdles of an academic career. Indeed, we observed faculty who had participated in ≥1 year of research during GME were less likely to leave academic medicine. It may be that GME programs and institutions offering research opportunities to trainees can better mentor trainees for successful academic careers more generally.

Given that grant awards and publications are known “traditional criteria” for promotion in academia [60], our findings that faculty who received RPGs and other types of grant funding were less likely to leave academic medicine were not surprising. URiM faculty are reported to have fewer publications [2] and lower scholarly productivity in higher-impact journals [61], receive fewer R01 grants [61–63], and have lower rates of promotion to rank of professor compared to White faculty [2]. Racial, ethnic, and sex differences in research funding may be at least partially responsible for the underrepresentation of certain groups of academic-medicine faculty at senior rank. Having fewer refereed publications also was reported to be associated with women’s lower likelihood of academic-medicine promotion and retention [64]. Institutions might consider research-focused faculty-development programs to help strengthen faculty grant-writing skills [40], foster productive mentor-mentee relationships [40,65], and offer funding opportunities to allow early-career faculty protected time for research. At one institution, modest one-year intramural grants awarded to junior faculty were associated with higher numbers of subsequent external grant awards and peer-reviewed publications among recipients [66], although potential bias introduced by the program’s competitive selection process cannot be discounted.

### Faculty department, track, and rank

We also observed differences in faculty attrition at the department level, with faculty first appointed in each of Family Medicine and Psychiatry departments more likely to leave academic medicine before reaching senior rank. Family Medicine and Psychiatry departments might offer less support for faculty to engage in research and scholarly productivity than Medicine and other departments offer. Family Medicine department chairs [67] and faculty [68] have described barriers to research and scholarly productivity, including having insufficient numbers of faculty engaged in research [68], serving as principal investigators, in addition to a lack of research-funding sources [67]. Psychiatry residents with greater financial debt have reported lower levels of interest in research [69]. In addition, a faculty-survey study reported an association between faculty members’ perceived lack of recognition of exemplary clinical service during promotion and tenure reviews and their intent to leave academic medicine [70]. We speculate that greater support for clinical service and teaching, especially in non-research-focused clinical departments at research-intensive institutions could help reduce attrition in such departments.

Faculty first appointed as instructors and at institutions where tenure was not available were also more likely to leave academic medicine before promotion to senior rank. Others have reported greater attrition among faculty appointed to the clinician-educator track, in particular [71]; notably, an earlier study of 83 medical schools reported higher proportions of female than male full-time faculty were on the clinician-educator track [72]. The Faculty Roster does not distinguish between faculty on the clinician-educator track and other non-tenure-eligible tracks, although we did not observe a significant difference in attrition between faculty on the tenure-eligible track and those on the non-tenure-eligible track. Notably, tenure tracks are an expensive venture for academic medicine [73] and are decreasing in number across U.S. medical schools [74].

### Strengths and limitations

The study’s strengths include having data for an entire national cohort of U.S. medical students who graduated from LCME-accredited medical schools with long-term follow-up after first full-time faculty appointment as instructor or assistant professor. We also had data for receipt of federal grant awards before attrition or promotion to associate professor and for institutional-level data indicating whether faculty were first appointed at research-intensive medical schools. As an individual’s demographic characteristics cannot be changed, we sought to identify other variables associated with attrition that can be targeted for intervention to increase the size and diversity of physicians at senior ranks in the academic-medicine workforce.

Our study also has limitations. Unmeasured variables, such as number of publications, known to be positively associated with faculty promotion and retention [64], characteristics of the institution where faculty completed GME, and availability of departmental support (e.g., protected time for research, mentoring, and other resources) for trainees and faculty, also might be associated with faculty attrition before reaching senior rank. We could not adjust our regression models for reasons why faculty left academic medicine, including specialty-based differences in salary, call schedules, and other factors that are likely to make academic careers challenging. Differences in compensation by sex or other characteristics may be relevant, as previously reported [75]. Physicians’ reasons for leaving the faculty may differ both within and across departments; however, we lacked data for their reasons. Furthermore, we did not have data about the disparate burden of parenting children, usually placed on mothers, nor about faculty perceptions of work-life balance to measure these factors in association with attrition from the faculty, as suggested by the literature [76–78]. We also acknowledge the potential for collinearity in our regression model, noting that several variables included in our model have been shown in other studies to be associated with each other. For example, female and URiM medical students were more likely to report diminished plans to pursue a career in academic-medicine from matriculation to graduation [43]. Nevertheless, we observed *independent effects* of individual- and institutional-level variables on faculty attrition prior to reaching senior rank, and the 95% CIs were reasonably small, indicating stable and reliable observations. That said, as one might expect, the power of the Hosmer-Lemeshow goodness-of-fit test increases with very large samples [79]. With over 30,000 cases included in this large national cohort, it was not surprising that the goodness-of-fit test was significant. Nevertheless, a significant Hosmer-Lemeshow test, given such a large sample, does *not* necessarily mean that our findings and variables we identified as significantly associated with attrition are neither sound nor useful [80]. In this study, we aimed to identify factors on which we might intervene to prevent attrition from academic medicine before attaining senior rank and succeeded in doing so. In addition, we cannot infer causation from our regression model identifying factors associated with faculty attrition. Our results may not be generalizable to faculty at non-LCME-accredited, osteopathic, or international medical schools. Our sample was limited to full-time faculty initially appointed as instructor or assistant professor, which represents most first-time faculty appointments. Thus, we cannot generalize our findings to faculty first appointed to part-time or volunteer positions or at the rank of associate or full professor; these faculty would be expected to have quite different experiences prior to appointment and were therefore excluded from sample selection.

## Conclusions

Nevertheless, we present new findings about individual- and institutional-level variables associated with attrition before promotion to senior rank, many of which are amenable to intervention. Professional-development interventions that foster students’ and trainees’ interest in academic-medicine careers, reduce debt, and promote grant-writing skills are needed to help reduce junior-faculty attrition, thereby increasing the number of faculty who are promoted to senior rank and retained in academic medicine. There is a critical need for professional-development opportunities that focus not only on supporting research and scholarly productivity, if required for promotion, but also supporting faculty on clinician-educator tracks, as these faculty, too, are essential to training future generations of physicians, in general, and building a more broadly experienced academic and biomedical-research workforce, in particular.

## Supporting information

S1 File Regression Analysis. (DOCX)

## References

[1] Fang D, Moy E, Colburn L, Hurley J. Racial and ethnic disparities in faculty promotion in academic medicine. JAMA. 2000;284(9):1085–92. doi: 10.1001/jama.284.9.1085 10974686

[2] Kaplan SE, Raj A, Carr PL, Terrin N, Breeze JL, Freund KM. Race/Ethnicity and Success in Academic Medicine: Findings From a Longitudinal Multi-Institutional Study. Acad Med. 2018;93(4):616–22. 29068820 10.1097/ACM.0000000000001968 PMC5916738

[3] Clark L, Shergina E, Machado N, Scheuermann TS, Sultana N, Polineni D, et al. Race and Ethnicity, Gender, and Promotion of Physicians in Academic Medicine. JAMA Network Open. 2024;7(11):e2446018. 10.1001/jamanetworkopen.2024.46018 PMC12262150 39602123

[4] Jeffe DB, Yan Y, Andriole DA. Competing Risks Analysis of Promotion and Attrition in Academic Medicine: A National Study of U.S. Medical School Graduates. Acad Med. 2019;94(2):227–36. doi: 10.1097/ACM.0000000000002441 30188371 PMC6351173

[5] Lett E, Orji WU, Sebro R. Declining racial and ethnic representation in clinical academic medicine: A longitudinal study of 16 US medical specialties. PLoS One. 2018;13(11):e0207274. doi: 10.1371/journal.pone.0207274 30444928 PMC6239326

[6] Day CS, Lage DE, Ahn CS. Diversity based on race, ethnicity, and sex between academic orthopaedic surgery and other specialties: a comparative study. J Bone Joint Surg Am. 2010;92(13):2328–35. doi: 10.2106/JBJS.I.01482 20926728

[7] Yu PT, Parsa PV, Hassanein O, Rogers SO, Chang DC. Minorities struggle to advance in academic medicine: A 12-y review of diversity at the highest levels of America’s teaching institutions. J Surg Res. 2013;182(2):212–8. doi: 10.1016/j.jss.2012.06.049 23582226

[8] Richter KP, Clark L, Wick JA, Cruvinel E, Durham D, Shaw P. Women physicians and promotion in academic medicine. N Engl J Med. 2020;383(22):2148–57. 10.1056/NEJMsa1916935. 33252871

[9] Breyer BN, Butler C, Fang R, Meeks W, Porten SP, North AC, et al. Promotion Disparities in Academic Urology. Urology. 2020;138:16–23. 10.1016/j.urology.2019.10.042 31917291

[10] Wooding DJ, Das P, Tiwana S, Siddiqi J, Khosa F. Race, ethnicity, and gender in academic obstetrics and gynecology: 12-year trends. Am J Obstet Gynecol MFM. 2020;2(4):100178. doi: 10.1016/j.ajogmf.2020.100178 33345906

[11] Linden JA, Baird J, Madsen TE, Rounds K, Lall MD, Raukar NP. Diversity of leadership in academic emergency medicine: Are we making progress? American J Emergency Medicine. 2022;57:6–13. 10.1016/j.ajem.2022.04.009 35462120

[12] Scheuermann TS, Clark L, Sultana N, Machado N, Shergina E, Polineni D. Race, Gender, and Faculty Retention in Academic Medicine. JAMA Network Open. 2024;7(11):e2445143. 10.1001/jamanetworkopen.2024.45143 PMC11565262 39541115

[13] Alam HB. Promotion. Clin Colon Rectal Surg. 2013;26(4):232–8. 10.1055/s-0033-1356723 24436683 PMC3835443

[14] Gonzalo JD, Dekhtyar M, Caverzagie KJ, Grant BK, Herrine SK, Nussbaum AM, et al. The triple helix of clinical, research, and education missions in academic health centers: A qualitative study of diverse stakeholder perspectives. Learn Health Syst. 2020;5(4):e10250. doi: 10.1002/lrh2.10250 34667874 PMC8512738

[15] Chapman T, Maxfield CM, Iyer RS. Promotion in academic radiology: context and considerations. Pediatr Radiol. 2023;53(1):8–11. doi: 10.1007/s00247-022-05535-z 36255458 PMC9579532

[16] Association of American Medical Colleges. Faculty Roster: U.S. Medical School Faculty, 2023 Report. Table 4: U.S. Medical School Faculty by Rank and Department, 2023. https://www.aamc.org/data-reports/faculty-institutions/report/faculty-roster-us-medical-school-faculty. Accessed May 24, 2026.

[17] Chen YW, Orlas C, Kim T, Chang DC, Kelleher CM. Workforce attrition among male and female physicians working in US academic hospitals, 2014-2019. JAMA Network Open. 2023;6(7):e2323872. doi: 10.1001/jamanetworkopen.2023.23872 PMC10352856 37459094

[18] von Elm E, Altman DG, Egger M, Pocock SJ, Gøtzsche PC, Vandenbroucke JP, et al. Strengthening the Reporting of Observational Studies in Epidemiology (STROBE) statement: guidelines for reporting observational studies. BMJ. 2007;335(7624):806–8. doi: 10.1136/bmj.39335.541782.AD 17947786 PMC2034723

[19] Association of American Medical Colleges. American Medical College Application Service® (AMCAS®). https://students-residents.aamc.org/applying-medical-school/article/apply-to-med-school-with-amcas/. Accessed May 24, 2026.

[20] Association of American Medical Colleges. Matriculating Student Questionnaire (MSQ). https://www.aamc.org/data-reports/students-residents/report/matriculating-student-questionnaire-msq. Accessed May 24, 2026.

[21] Association of American Medical Colleges. Graduation Questionnaire (GQ). https://www.aamc.org/data-reports/students-residents/report/graduation-questionnaire-gq. Accessed May 24, 2026.

[22] Association of American Medical Colleges. GME Track. Available at: https://www.aamc.org/data-reports/students-residents/report/gme-track. Accessed May 24, 2026.

[23] Lauer M, D R. Analyses of Demographic-Specific Funding Rates for Type 1 Research Project Grant and R01-Equivalent Applications. 2023. https://report.nih.gov/sites/report/files/docs/RPG_demog_3_13_23_long_version.pdf. Accessed May 24, 2026.

[24] Association of American Medical Colleges. U.S. Medical School Revenues. https://www.aamc.org/data-reports/faculty-institutions/report/us-medical-school-revenues#:~:text=The%20U.S.%20Medical%20School%20Revenue,provisional%2C%20or%20full%20LCME%20accreditation. Accessed May 24, 2026.

[25] Association of American Medical Colleges AAMC. AAMC. Faculty Roster. https://www.aamc.org/data-reports/faculty-institutions/faculty-roster. Accessed May 24, 2026.

[26] Jagsi R, Griffith KA, Stewart A, Sambuco D, DeCastro R, Ubel PA. Gender differences in the salaries of physician researchers. JAMA. 2012;307(22):2410–7. doi: 10.1001/jama.2012.6183 22692173

[27] Jena AB, Olenski AR, Blumenthal DM. Sex Differences in Physician Salary in US Public Medical Schools. JAMA Intern Med. 2016;176(9):1294–304. doi: 10.1001/jamainternmed.2016.3284 27400435 PMC5558151

[28] Owda D, Mensah MO, Yang D, Canavan ME, Gross CP, Chaudhry SI. Salary Differences by Gender, Race, and Ethnicity Among Assistant Professors at US Medical Schools. JAMA Netw Open. 2025;8(5):e259583. doi: 10.1001/jamanetworkopen.2025.9583 40366659 PMC12079292

[29] Ahn DJ, Garg N, Naik AG, Fan J, Wei H, Song BB, et al. Where Do I Fit In? A Perspective on Challenges Faced by Asian American Medical Students. Health Equity. 2021;5(1):324–8. doi: 10.1089/heq.2020.0158 34036216 PMC8140354

[30] Choi AMK, Rustgi AK. Diversity in leadership at academic medical centers: addressing underrepresentation among Asian American faculty. JAMA. 2021;326(7):605–6. 34402820 10.1001/jama.2021.12028

[31] Venkataraman S, Nguyen M, Boatright D. Barriers to Advancement-Unequal Opportunities in Academic Promotion Based on Race, Ethnicity, and Gender. JAMA Netw Open. 2024;7(11):e2445971. doi: 10.1001/jamanetworkopen.2024.45971 39602128

[32] Cohen JG, Sherman AE, Kiet TK, Kapp DS, Osann K, Chen L, et al. Characteristics of success in mentoring and research productivity - a case-control study of academic centers. Gynecol Oncol. 2012;125(1):8–13. doi: 10.1016/j.ygyno.2012.01.005 22252098

[33] Lee R, Lucas R, Dickerman J, Day LW, Guzman D, Kothari P, et al. Designing Prefaculty Competencies for Diverse Learners Through a Modified Delphi Process. JAMA Netw Open. 2024;7(7):e2424003. doi: 10.1001/jamanetworkopen.2024.24003 39058487 PMC11282442

[34] Bonifacino E, Ufomata EO, Farkas AH, Turner R, Corbelli JA. Mentorship of Underrepresented Physicians and Trainees in Academic Medicine: a Systematic Review. J Gen Intern Med. 2021;36(4):1023–34. doi: 10.1007/s11606-020-06478-7 33532959 PMC7852467

[35] Freeman WE, Sanders C, Hincapié A, Jeffe DB. Medical Student Factors Associated with Future Office-based Primary-care Practice. Journal of the American Board of Family Medicine. In press.

[36] Havemann C, Mason HRC, Russell RG, Casillas A, Nguyen M, Boatright D, et al. Challenges Facing First-Generation College Graduates in Medical School: A Qualitative Analysis. JAMA Netw Open. 2023;6(12):e2347528. doi: 10.1001/jamanetworkopen.2023.47528 38091039 PMC10719755

[37] Jean-Louis G, Ayappa I, Rapoport D, Zizi F, Airhihenbuwa C, Okuyemi K, et al. Mentoring junior URM scientists to engage in sleep health disparities research: experience of the NYU PRIDE Institute. Sleep Med. 2016;18:108–17. doi: 10.1016/j.sleep.2015.09.010 26631970 PMC4762758

[38] Pace BS, Makala LH, Sarkar R, Liu L, Takezaki M, Mohandas N, et al. Enhancing diversity in the hematology biomedical research workforce: A mentoring program to improve the odds of career success for early stage investigators. Am J Hematol. 2017;92(12):1275–9. doi: 10.1002/ajh.24899 28857249 PMC6312723

[39] Jeffe DB, Rice TK, Boyington JEA, Rao DC, Jean-Louis G, Dávila-Román VG, et al. Development and Evaluation of Two Abbreviated Questionnaires for Mentoring and Research Self-Efficacy. Ethn Dis. 2017;27(2):179–88. 10.18865/ed.27.2.179 . Accessed August 7, 2026 28439189 PMC5398177

[40] Boutjdir M, Aromolaran AS, de las Fuentes L, Boyington JEA, Arteaga SS, Jobe J, et al. Research Education and Mentoring Program in Cardiovascular Diseases for Under-Represented Junior Faculty From NHLBI SIPID/PRIDE. J Am Coll Cardiol. 2019;73(14):1861–5. doi: 10.1016/j.jacc.2019.01.042 30975303 PMC6464379

[41] Crites GE, Ward WL, Archuleta P, Fornari A, Hill SEM, Westervelt LM, et al. A scoping review of health care faculty mentorship programs in academia: implications for program design, implementation, and outcome evaluation. J Contin Educ Health Prof. 2023;43(1):42–51. 36215162 10.1097/CEH.0000000000000459

[42] Pfund C, House SC, Asquith P, Fleming MF, Buhr KA, Burnham EL, et al. Training mentors of clinical and translational research scholars: a randomized controlled trial. Acad Med. 2014;89(5):774–82. doi: 10.1097/ACM.0000000000000218 24667509 PMC4121731

[43] Jeffe DB, Andriole DA, Hageman HL, Whelan AJ. Reaping what we sow: the emerging academic medicine workforce. J Natl Med Assoc. 2008;100(9):1026–34. doi: 10.1016/s0027-9684(15)31439-5 18807430

[44] Haviland MG, Yamagata H, Werner LS, Zhang K, Dial TH, Sonne JL. Student mistreatment in medical school and planning a career in academic medicine. Teach Learn Med. 2011;23(3):231–7. doi: 10.1080/10401334.2011.586914 21745057

[45] Vetter MH, Carter M. Differences between first and fourth year medical students’ interest in pursuing careers in academic medicine. Int J Med Educ. 2016;7:154–7. doi: 10.5116/ijme.571b.af3d 27219295 PMC4885633

[46] Ortega G, Smith C, Pichardo MS, Ramirez A, Soto-Greene M, Sánchez JP. Preparing for an Academic Career: The Significance of Mentoring. MedEdPORTAL. 2018;14:10690. doi: 10.15766/mep_2374-8265.10690 30800890 PMC6342371

[47] Andriole DA, Yan Y, Jeffe DB. Mediators of racial/ethnic disparities in mentored K award receipt among U.S. medical school graduates. Acad Med. 2017;92(10):1440–8. 28767497 10.1097/ACM.0000000000001871 PMC5617780

[48] Jeffe DB, Andriole DA. Prevalence and predictors of US medical graduates’ federal F32, mentored-K, and R01 awards: a national cohort study. J Investig Med. 2018;66(2):340–50. doi: 10.1136/jim-2017-000515 28954846 PMC5964605

[49] Jeffe DB, Yan Y, Andriole DA. Do research activities during college, medical school, and residency mediate racial/ethnic disparities in full-time faculty appointments at U.S. Medical schools?. Acad Med. 2012;87(11):1582–93. doi: 10.1097/ACM.0b013e31826e3297 23018339 PMC3592974

[50] Andriole DA, Jeffe DB. The road to an academic medicine career: a national cohort study of male and female U.S. medical graduates. Acad Med. 2012;87(12):1722–33. doi: 10.1097/ACM.0b013e318271e57b 23095924 PMC3631320

[51] Andriole DA, Jeffe DB. Predictors of full-time faculty appointment among MD-PhD program graduates: a national cohort study. Med Educ Online. 2016;21:30941. doi: 10.3402/meo.v21.30941 27189673 PMC4870348

[52] Baimas-George M, Fleischer B, Korndorffer JR Jr, Slakey D, DuCoin C. The economics of private practice versus academia in surgery. J Surg Educ. 2018;75(5):1276–80. doi: 10.1016/j.jsurg.2018.03.006 29674107

[53] Phillips JP, Peterson LE, Fang B, Kovar-Gough I, Phillips RL Jr. Debt and the emerging physician workforce: the relationship between educational debt and family medicine residents’ practice and fellowship intentions. Acad Med. 2019;94(2):267–73. doi: 10.1097/ACM.0000000000002468 30256252

[54] Holaday LW, Weiss JM, Sow SD, Perez HR, Ross JS, Genao I. Differences in debt among postgraduate medical residents by self-designated race and ethnicity, 2014-19. Health Aff (Millwood). 2023;42(1):63–73. 10.1377/hlthaff.2022.00446 36623219 PMC9954659

[55] Garrison HH, Ley TJ. Physician-scientists in the United States at 2020: Trends and concerns. FASEB J. 2022;36(5):e22253. doi: 10.1096/fj.202200327 35349197 PMC9314812

[56] National Institutes of Health, National Institute of General Medical Sciences. Medical Scientist Training Program (MSTP) (T32). https://grants.nih.gov/grants/guide/pa-files/PAR-24-128.html. Accessed 2026 May 24.

[57] Jeffe DB, Andriole DA. A national cohort study of MD-PhD graduates of medical schools with and without funding from the National Institute of General Medical Sciences’ Medical Scientist Training Program. Acad Med. 2011;86(8):953–61. doi: 10.1097/ACM.0b013e31822225c5 21694566 PMC3145809

[58] Andriole DA, Jeffe DB, Hageman HL, Ephgrave K, Lypson ML, Mavis B, et al. Variables associated with full-time faculty appointment among contemporary U.S. Medical school graduates: implications for academic medicine workforce diversity. Acad Med. 2010;85(7):1250–7. doi: 10.1097/ACM.0b013e3181e10159 20592523 PMC3236355

[59] Barker JC, Jalilvand A, Onuma A, Shelby R, Shah K, Daulton R, et al. Facilitating Success of the Early Stage Surgeon Scientist Trainee: Growing the Surgeon Scientist Pipeline. Ann Surg. 2022;275(2):e334–44. doi: 10.1097/SLA.0000000000004924 33938494 PMC8977112

[60] Rice DB, Raffoul H, Ioannidis JPA, Moher D. Academic criteria for promotion and tenure in biomedical sciences faculties: cross sectional analysis of international sample of universities. BMJ. 2020;369:m2081. doi: 10.1136/bmj.m2081 32586791 PMC7315647

[61] Ginther DK, Basner J, Jensen U, Schnell J, Kington R, Schaffer WT. Publications as predictors of racial and ethnic differences in NIH research awards. PLoS One. 2018;13(11):e0205929. doi: 10.1371/journal.pone.0205929 30427864 PMC6235266

[62] Ginther DK, Haak LL, Schaffer WT, Kington R. Are race, ethnicity, and medical school affiliation associated with NIH R01 type 1 award probability for physician investigators? Acad Med. 2012;87(11):1516–24. doi: 10.1097/ACM.0b013e31826d726b 23018334 PMC3485449

[63] Ginther DK, Kahn S, Schaffer WT. Gender, Race/Ethnicity, and National Institutes of Health R01 Research Awards: Is There Evidence of a Double Bind for Women of Color? Acad Med. 2016;91(8):1098–107. doi: 10.1097/ACM.0000000000001278 27306969 PMC4965301

[64] Carr PL, Raj A, Kaplan SE, Terrin N, Breeze JL, Freund KM. Gender differences in academic medicine: retention, rank, and leadership comparisons from the national faculty survey. Acad Med. 2018;93(11):1694–9. 10.1097/acm.0000000000002146 29384751 PMC6066448

[65] Sandi G, Chubinskaya S. A faculty development model that promotes success of early career faculty in academic medicine. J Contin Educ Health Prof. 2020;40(1):69–72. 10.1097/ceh.0000000000000282 32149950

[66] Virdi AS, Sandi G, Chubinskaya S. Intramural Grant Program to Promote Research Activity Among Early-Career Faculty Members. Acad Med. 2022;97(9):1331–4. doi: 10.1097/ACM.0000000000004662 35263304

[67] Weidner A, Peterson LE, Mainous AG, Datta A, Ewigman B. The Current State of Research Capacity in US Family Medicine Departments. Fam Med. 2019;51(2):112–9. 10.22454/fammed.2019.180310 30736036

[68] Brocato JJ, Mavis B. The research productivity of faculty in family medicine departments at U.S. medical schools: a national study. Acad Med. 2005;80(3):244–52. doi: 10.1097/00001888-200503000-00008 15734806

[69] Silberman EK, Belitsky R, Bernstein CA, Cabaniss DL, Crisp-Han H, Dickstein LJ, et al. Recruiting researchers in psychiatry: the influence of residency vs. early motivation. Acad Psychiatry. 2012;36(2):85–90. doi: 10.1176/appi.ap.10010010 22532195

[70] Lowenstein SR, Fernandez G, Crane LA. Medical school faculty discontent: prevalence and predictors of intent to leave academic careers. BMC Med Educ. 2007;7:37. doi: 10.1186/1472-6920-7-37 17935631 PMC2194670

[71] Myers O, Vick K, Greenberg N, Sood A. Faculty retention at a school of medicine, 2010-2022. Chron Mentor Coach. 2023;7(Si16):388–93. 38187463 PMC10768923

[72] Mayer AP, Blair JE, Ko MG, Hayes SN, Chang Y-HH, Caubet SL, et al. Gender distribution of U.S. medical school faculty by academic track type. Acad Med. 2014;89(2):312–7. doi: 10.1097/ACM.0000000000000089 24362384

[73] Bhardwaj A. What’s new in academic International medicine? The evolving terrain of American academic medicine. Int J Acad Med. 2019;5(2):85. doi: 10.4103/ijam.ijam_25_19

[74] Mallon WT, Cox N. Promotion and tenure policies and practices at U.S. medical schools: is tenure irrelevant or more relevant than ever? Acad Med. 2024;99(7):724–32. 10.1097/acm.0000000000005689 38489477

[75] Bucklin BA, Valley M, Welch C, Tran ZV, Lowenstein SR. Predictors of early faculty attrition at one Academic Medical Center. BMC Med Educ. 2014;14:27. doi: 10.1186/1472-6920-14-27 24512629 PMC3923102

[76] Juengst SB, Royston A, Huang I, Wright B. Family Leave and Return-to-Work Experiences of Physician Mothers. JAMA Netw Open. 2019;2(10):e1913054. 10.1001/jamanetworkopen.2019.13054 PMC6804274 31603485

[77] Thompson-Burdine JA, Telem DA, Waljee JF, Newman EA, Coleman DM, Stoll HI, et al. Defining Barriers and Facilitators to Advancement for Women in Academic Surgery. JAMA Netw Open. 2019;2(8):e1910228. doi: 10.1001/jamanetworkopen.2019.10228 31469392 PMC6724152

[78] Messman A, Stansfield RB, Liu Y, Collins J, Matthews M, Ehrman R. Identifying positive and negative factors that affect the promotion of clinical faculty at the Wayne State University School of Medicine: does gender matter? Cureus. 2022;14(10):e29954. 10.7759/cureus.29954 PMC9635576 36348900

[79] Paul P, Pennell ML, Lemeshow S. Standardizing the power of the Hosmer-Lemeshow goodness of fit test in large data sets. Stat Med. 2013;32(1):67–80. doi: 10.1002/sim.5525 22833304

[80] Kramer AA, Zimmerman JE. Assessing the calibration of mortality benchmarks in critical care: The Hosmer-Lemeshow test revisited. Crit Care Med. 2007;35(9):2052–6. doi: 10.1097/01.CCM.0000275267.64078.B0 17568333

