# Supplementary material for: Factors associated with academic-medicine faculty attrition before promotion to senior rank: A national-cohort study
Source: PLoS One. 2026 Sep 2;21(9):e0355830. doi: 10.1371/journal.pone.0355830 (PMC13537596; doi:10.1371/journal.pone.0355830)
Supplement: Supplementary file 1 [file pone.0355830.s001.docx]

**Logistic Regression**

| **Case Processing Summary** | | | |
| --- | --- | --- | --- |
| Unweighted Cases^a^ | | N | Percent |
| Selected Cases | Included in Analysis | 34478 | 100.0 |
|  | Missing Cases | 0 | .0 |
|  | Total | 34478 | 100.0 |
| Unselected Cases | | 0 | .0 |
| Total | | 34478 | 100.0 |
| a. If weight is in effect, see classification table for the total number of cases. | | | |

| **Dependent Variable Encoding** | |
| --- | --- |
| Original Value | Internal Value |
| .00 Retained with or without promotion to assoc or full prof | 0 |
| 1.00 Attrition before promotion to assoc or full prof | 1 |

| **Categorical Variables Codings** | | | | | | | | | | |
| --- | --- | --- | --- | --- | --- | --- | --- | --- | --- | --- |
|  | | Frequency | Parameter coding | | | | | | | |
|  |  |  | (1) | (2) | (3) | (4) | (5) | (6) | (7) | (8) |
| NEW20_SUBUNIT1_r5 Department Subunit for first appointment, 9 category | 1.00 Internal Medicine | 9235 | .000 | .000 | .000 | .000 | .000 | .000 | .000 | .000 |
|  | 2.00 Family Medicine | 1269 | 1.000 | .000 | .000 | .000 | .000 | .000 | .000 | .000 |
|  | 3.00 Pediatrics | 5097 | .000 | 1.000 | .000 | .000 | .000 | .000 | .000 | .000 |
|  | 4.00 OB/GYN | 1820 | .000 | .000 | 1.000 | .000 | .000 | .000 | .000 | .000 |
|  | 5.00 Surgery (including Ortho, Ophtho, Oto) | 6189 | .000 | .000 | .000 | 1.000 | .000 | .000 | .000 | .000 |
|  | 6.00 Psychiatry | 1768 | .000 | .000 | .000 | .000 | 1.000 | .000 | .000 | .000 |
|  | 7.00 Neurology | 964 | .000 | .000 | .000 | .000 | .000 | 1.000 | .000 | .000 |
|  | 8.00 Facilities-based (Anes, EM, Path, Rads) | 6882 | .000 | .000 | .000 | .000 | .000 | .000 | 1.000 | .000 |
|  | 9.00 All Other Specialties | 1254 | .000 | .000 | .000 | .000 | .000 | .000 | .000 | 1.000 |
| GQdebt_6cat_sysmis_r2024 recoded with fewer categories Direct copy of GQ_total_debt_cat_rsysmis for Yan; total debt indicated on GQ, sysmis included | .00 No debt ($0) | 5304 | .000 | .000 | .000 |  |  |  |  |  |
|  | 1.00 $1 to $99,999 | 12013 | 1.000 | .000 | .000 |  |  |  |  |  |
|  | 2.00 $100,000 more | 10908 | .000 | 1.000 | .000 |  |  |  |  |  |
|  | 99.00 sysmis | 6253 | .000 | .000 | 1.000 |  |  |  |  |  |
| Tenure1_sysmis Direct copy of new20TENURE_CD1_rsysmis for Yan; Tenure status at first appointment | 2 NA_TRACK = not tenured, not on tenure eligible track | 23733 | .000 | .000 | .000 |  |  |  |  |  |
|  | 3 NO_TENURE = not tenured, on tenure eligible track | 5487 | 1.000 | .000 | .000 |  |  |  |  |  |
|  | 4 NOT_AVAIL = tenure not available at that school | 2183 | .000 | 1.000 | .000 |  |  |  |  |  |
|  | 5 Sysmis | 3075 | .000 | .000 | 1.000 |  |  |  |  |  |
| RGrant_Assoc_3catNEW2023 new 2023 combining R grants into 1 group after graduation, but before promotion to associate professor or before attrition from academia | .00 No, never had any grant in this time frame | 31935 | .000 | .000 |  |  |  |  |  |  |
|  | 1.00 Had R01 or other RPG grants | 616 | 1.000 | .000 |  |  |  |  |  |  |
|  | 2.00 No had only non-RPG grants (both pre- and post-doctoral; includes R13 and R36) | 1927 | .000 | 1.000 |  |  |  |  |  |  |
| AppMatRace_r_2023URM race ethnicity 2023 with corrected URM groups | 1.00 White | 23119 | .000 | .000 |  |  |  |  |  |  |
|  | 2.00 Asian | 7002 | 1.000 | .000 |  |  |  |  |  |  |
|  | 3.00 URM | 4357 | .000 | 1.000 |  |  |  |  |  |  |
| MSQ_parent_educ_r2_r3 recoded MSQ_parent_educ_r2 continuing generation college grads as a ref group | 1.00 first gen college grads | 5120 | 1.000 | .000 |  |  |  |  |  |  |
|  | 2.00 both parents education unknown | 2427 | .000 | 1.000 |  |  |  |  |  |  |
|  | 3.00 continuing college grads | 26931 | .000 | .000 |  |  |  |  |  |  |
| GQCareerPref_sysmis Direct copy of NEW_GQ_CAREER_PREF_CAT_faculty_r_sysmis for Yan; Career preference indicated on GQ, 3category including sysmis | .00 clin practice, other (incl. nonuniv research scientist), undecided | 15559 | 1.000 | .000 |  |  |  |  |  |  |
|  | 1.00 sysmis | 6062 | .000 | 1.000 |  |  |  |  |  |  |
|  | 2.00 FT Faculty in basic and clinical sciences | 12857 | .000 | .000 |  |  |  |  |  |  |
| MED_SCHOOL_TOP40_r Is med school a top 40 research institution? based on 2007-2008 all federal research expenditures | 0 no | 20173 | .000 |  |  |  |  |  |  |  |
|  | 1 yes | 14305 | 1.000 |  |  |  |  |  |  |  |
| FR_Top40_r recoding into ever vs never the Direct copy of FacultyRosterNew20Top40 for Yan; Top40 Status for first appointment institution | .00 Never a top40 | 11125 | .000 |  |  |  |  |  |  |  |
|  | 1.00 Ever a top40 | 23353 | 1.000 |  |  |  |  |  |  |  |
| new20RANK_CD1 RANK_CD new20 | 3 assistant professor | 22966 | .000 |  |  |  |  |  |  |  |
|  | 4 instructor | 11512 | 1.000 |  |  |  |  |  |  |  |
| GME19_GME_Research_YN Did trainee ever complete a research year during GME? | .00 trainee never completed a research year during GME | 27276 | .000 |  |  |  |  |  |  |  |
|  | 1.00 trainee completed at least 1 year of research during GME | 7202 | 1.000 |  |  |  |  |  |  |  |
| APP_MAT_SEX_r gender | 1 female | 15546 | 1.000 |  |  |  |  |  |  |  |
|  | 2 male | 18932 | .000 |  |  |  |  |  |  |  |

**Block 0: Beginning Block**

| **Classification Table**^a,b^ | | | | | |
| --- | --- | --- | --- | --- | --- |
|  | Observed | | Predicted | | |
|  |  |  | status_Assoc_WF_r Recoded into 2 groups Status variable for attrition vs retention | | Percentage Correct |
|  |  |  | .00 Retained with or without promotion to assoc or full prof | 1.00 Attrition before promotion to assoc or full prof |  |
| Step 0 | status_Assoc_WF_r Recoded into 2 groups Status variable for attrition vs retention | .00 Retained with or without promotion to assoc or full prof | 20336 | 0 | 100.0 |
|  |  | 1.00 Attrition before promotion to assoc or full prof | 14142 | 0 | .0 |
|  | Overall Percentage | |  |  | 59.0 |
| a. Constant is included in the model. | | | | | |
| b. The cut value is .500 | | | | | |

| **Variables in the Equation** | | | | | | | |
| --- | --- | --- | --- | --- | --- | --- | --- |
|  | | B | S.E. | Wald | df | Sig. | Exp(B) |
| Step 0 | Constant | -.363 | .011 | 1100.602 | 1 | <.001 | .695 |

| **Variables not in the Equation** | | | | | |
| --- | --- | --- | --- | --- | --- |
|  | | | Score | df | Sig. |
| Step 0 | Variables | months to status2_WFversion outcome | 8398.985 | 1 | <.001 |
|  |  | gender(1) | 81.963 | 1 | <.001 |
|  |  | race ethnicity 2023 with corrected URM groups | 100.306 | 2 | <.001 |
|  |  | race ethnicity 2023 with corrected URM groups(1) | .266 | 1 | .606 |
|  |  | race ethnicity 2023 with corrected URM groups(2) | 94.417 | 1 | <.001 |
|  |  | recoded MSQ_parent_educ_r2 continuing generation college grads as a ref group | 71.470 | 2 | <.001 |
|  |  | recoded MSQ_parent_educ_r2 continuing generation college grads as a ref group(1) | 69.581 | 1 | <.001 |
|  |  | recoded MSQ_parent_educ_r2 continuing generation college grads as a ref group(2) | .166 | 1 | .684 |
|  |  | Is med school a top 40 research institution? based on 2007-2008 all federal research expenditures(1) | 297.036 | 1 | <.001 |
|  |  | Direct copy of NEW_GQ_CAREER_PREF_CAT_faculty_r_sysmis for Yan; Career preference indicated on GQ, 3category including sysmis | 490.725 | 2 | <.001 |
|  |  | Direct copy of NEW_GQ_CAREER_PREF_CAT_faculty_r_sysmis for Yan; Career preference indicated on GQ, 3category including sysmis(1) | 397.481 | 1 | <.001 |
|  |  | Direct copy of NEW_GQ_CAREER_PREF_CAT_faculty_r_sysmis for Yan; Career preference indicated on GQ, 3category including sysmis(2) | .174 | 1 | .676 |
|  |  | recoded with fewer categories Direct copy of GQ_total_debt_cat_rsysmis for Yan; total debt indicated on GQ, sysmis included | 78.954 | 3 | <.001 |
|  |  | recoded with fewer categories Direct copy of GQ_total_debt_cat_rsysmis for Yan; total debt indicated on GQ, sysmis included(1) | .780 | 1 | .377 |
|  |  | recoded with fewer categories Direct copy of GQ_total_debt_cat_rsysmis for Yan; total debt indicated on GQ, sysmis included(2) | 48.505 | 1 | <.001 |
|  |  | recoded with fewer categories Direct copy of GQ_total_debt_cat_rsysmis for Yan; total debt indicated on GQ, sysmis included(3) | .228 | 1 | .633 |
|  |  | Did trainee ever complete a research year during GME?(1) | 487.894 | 1 | <.001 |
|  |  | RANK_CD new20(1) | 798.445 | 1 | <.001 |
|  |  | Direct copy of new20TENURE_CD1_rsysmis for Yan; Tenure status at first appointment | 375.159 | 3 | <.001 |
|  |  | Direct copy of new20TENURE_CD1_rsysmis for Yan; Tenure status at first appointment(1) | 48.899 | 1 | <.001 |
|  |  | Direct copy of new20TENURE_CD1_rsysmis for Yan; Tenure status at first appointment(2) | 8.433 | 1 | .004 |
|  |  | Direct copy of new20TENURE_CD1_rsysmis for Yan; Tenure status at first appointment(3) | 339.623 | 1 | <.001 |
|  |  | new 2023 combining R grants into 1 group after graduation, but before promotion to associate professor or before attrition from academia | 817.567 | 2 | <.001 |
|  |  | new 2023 combining R grants into 1 group after graduation, but before promotion to associate professor or before attrition from academia(1) | 248.376 | 1 | <.001 |
|  |  | new 2023 combining R grants into 1 group after graduation, but before promotion to associate professor or before attrition from academia(2) | 544.181 | 1 | <.001 |
|  |  | Department Subunit for first appointment, 9 category | 416.280 | 8 | <.001 |
|  |  | Department Subunit for first appointment, 9 category(1) | 88.189 | 1 | <.001 |
|  |  | Department Subunit for first appointment, 9 category(2) | 82.625 | 1 | <.001 |
|  |  | Department Subunit for first appointment, 9 category(3) | 9.663 | 1 | .002 |
|  |  | Department Subunit for first appointment, 9 category(4) | 87.342 | 1 | <.001 |
|  |  | Department Subunit for first appointment, 9 category(5) | 65.321 | 1 | <.001 |
|  |  | Department Subunit for first appointment, 9 category(6) | 25.753 | 1 | <.001 |
|  |  | Department Subunit for first appointment, 9 category(7) | 106.188 | 1 | <.001 |
|  |  | Department Subunit for first appointment, 9 category(8) | 3.871 | 1 | .049 |
|  |  | recoding into ever vs never the Direct copy of FacultyRosterNew20Top40 for Yan; Top40 Status for first appointment institution(1) | 174.988 | 1 | <.001 |
|  | Overall Statistics | | 11045.664 | 28 | <.001 |

**Block 1: Method = Enter**

| **Omnibus Tests of Model Coefficients** | | | | |
| --- | --- | --- | --- | --- |
|  | | Chi-square | df | Sig. |
| Step 1 | Step | 12978.971 | 28 | <.001 |
|  | Block | 12978.971 | 28 | <.001 |
|  | Model | 12978.971 | 28 | <.001 |

| **Model Summary** | | | |
| --- | --- | --- | --- |
| Step | -2 Log likelihood | Cox & Snell R Square | Nagelkerke R Square |
| 1 | 33698.865^a^ | .314 | .423 |
| a. Estimation terminated at iteration number 5 because parameter estimates changed by less than .001. | | | |

| **Hosmer and Lemeshow Test** | | | |
| --- | --- | --- | --- |
| Step | Chi-square | df | Sig. |
| 1 | 604.830 | 8 | <.001 |

| **Contingency Table for Hosmer and Lemeshow Test** | | | | | | |
| --- | --- | --- | --- | --- | --- | --- |
|  | | status_Assoc_WF_r Recoded into 2 groups Status variable for attrition vs retention = .00 Retained with or without promotion to assoc or full prof | | status_Assoc_WF_r Recoded into 2 groups Status variable for attrition vs retention = 1.00 Attrition before promotion to assoc or full prof | | Total |
|  |  | Observed | Expected | Observed | Expected |  |
| Step 1 | 1 | 3114 | 3308.482 | 334 | 139.518 | 3448 |
|  | 2 | 2991 | 3105.417 | 457 | 342.583 | 3448 |
|  | 3 | 2855 | 2892.205 | 593 | 555.795 | 3448 |
|  | 4 | 2766 | 2659.909 | 682 | 788.091 | 3448 |
|  | 5 | 2569 | 2399.197 | 879 | 1048.803 | 3448 |
|  | 6 | 2317 | 2073.094 | 1131 | 1374.906 | 3448 |
|  | 7 | 1816 | 1669.586 | 1632 | 1778.414 | 3448 |
|  | 8 | 1149 | 1190.906 | 2299 | 2257.094 | 3448 |
|  | 9 | 612 | 722.434 | 2836 | 2725.566 | 3448 |
|  | 10 | 147 | 314.769 | 3299 | 3131.231 | 3446 |

| **Classification Table**^a^ | | | | | |
| --- | --- | --- | --- | --- | --- |
|  | Observed | | Predicted | | |
|  |  |  | status_Assoc_WF_r Recoded into 2 groups Status variable for attrition vs retention | | Percentage Correct |
|  |  |  | .00 Retained with or without promotion to assoc or full prof | 1.00 Attrition before promotion to assoc or full prof |  |
| Step 1 | status_Assoc_WF_r Recoded into 2 groups Status variable for attrition vs retention | .00 Retained with or without promotion to assoc or full prof | 17424 | 2912 | 85.7 |
|  |  | 1.00 Attrition before promotion to assoc or full prof | 4604 | 9538 | 67.4 |
|  | Overall Percentage | |  |  | 78.2 |
| a. The cut value is .500 | | | | | |

| **Variables in the Equation** | | | | | | | | | |
| --- | --- | --- | --- | --- | --- | --- | --- | --- | --- |
|  | | B | S.E. | Wald | df | Sig. | Exp(B) | 95% C.I.for EXP(B) | |
|  |  |  |  |  |  |  |  | Lower | Upper |
| Step 1^a^ | months to status2_WFversion outcome | -.030 | .000 | 6526.191 | 1 | <.001 | .970 | .969 | .971 |
|  | gender(1) | .249 | .028 | 78.331 | 1 | <.001 | 1.283 | 1.214 | 1.356 |
|  | race ethnicity 2023 with corrected URM groups |  |  | 48.164 | 2 | <.001 |  |  |  |
|  | race ethnicity 2023 with corrected URM groups(1) | .117 | .034 | 11.660 | 1 | <.001 | 1.125 | 1.051 | 1.203 |
|  | race ethnicity 2023 with corrected URM groups(2) | .273 | .042 | 42.938 | 1 | <.001 | 1.313 | 1.211 | 1.425 |
|  | recoded MSQ_parent_educ_r2 continuing generation college grads as a ref group |  |  | 21.063 | 2 | <.001 |  |  |  |
|  | recoded MSQ_parent_educ_r2 continuing generation college grads as a ref group(1) | .171 | .039 | 19.616 | 1 | <.001 | 1.186 | 1.100 | 1.279 |
|  | recoded MSQ_parent_educ_r2 continuing generation college grads as a ref group(2) | -.036 | .053 | .452 | 1 | .501 | .965 | .870 | 1.071 |
|  | Is med school a top 40 research institution? based on 2007-2008 all federal research expenditures(1) | -.273 | .029 | 89.974 | 1 | <.001 | .761 | .719 | .805 |
|  | Direct copy of NEW_GQ_CAREER_PREF_CAT_faculty_r_sysmis for Yan; Career preference indicated on GQ, 3category including sysmis |  |  | 97.315 | 2 | <.001 |  |  |  |
|  | Direct copy of NEW_GQ_CAREER_PREF_CAT_faculty_r_sysmis for Yan; Career preference indicated on GQ, 3category including sysmis(1) | .289 | .031 | 85.525 | 1 | <.001 | 1.334 | 1.255 | 1.419 |
|  | Direct copy of NEW_GQ_CAREER_PREF_CAT_faculty_r_sysmis for Yan; Career preference indicated on GQ, 3category including sysmis(2) | .491 | .104 | 22.160 | 1 | <.001 | 1.634 | 1.332 | 2.004 |
|  | recoded with fewer categories Direct copy of GQ_total_debt_cat_rsysmis for Yan; total debt indicated on GQ, sysmis included |  |  | 36.797 | 3 | <.001 |  |  |  |
|  | recoded with fewer categories Direct copy of GQ_total_debt_cat_rsysmis for Yan; total debt indicated on GQ, sysmis included(1) | .144 | .042 | 11.765 | 1 | <.001 | 1.155 | 1.064 | 1.254 |
|  | recoded with fewer categories Direct copy of GQ_total_debt_cat_rsysmis for Yan; total debt indicated on GQ, sysmis included(2) | .187 | .043 | 19.189 | 1 | <.001 | 1.206 | 1.109 | 1.312 |
|  | recoded with fewer categories Direct copy of GQ_total_debt_cat_rsysmis for Yan; total debt indicated on GQ, sysmis included(3) | -.295 | .107 | 7.642 | 1 | .006 | .744 | .604 | .918 |
|  | Did trainee ever complete a research year during GME?(1) | -.370 | .036 | 105.515 | 1 | <.001 | .691 | .643 | .741 |
|  | RANK_CD new20(1) | 1.122 | .031 | 1297.100 | 1 | <.001 | 3.072 | 2.890 | 3.265 |
|  | Direct copy of new20TENURE_CD1_rsysmis for Yan; Tenure status at first appointment |  |  | 90.301 | 3 | <.001 |  |  |  |
|  | Direct copy of new20TENURE_CD1_rsysmis for Yan; Tenure status at first appointment(1) | .050 | .038 | 1.739 | 1 | .187 | 1.051 | .976 | 1.133 |
|  | Direct copy of new20TENURE_CD1_rsysmis for Yan; Tenure status at first appointment(2) | .248 | .055 | 20.195 | 1 | <.001 | 1.281 | 1.150 | 1.427 |
|  | Direct copy of new20TENURE_CD1_rsysmis for Yan; Tenure status at first appointment(3) | .434 | .050 | 76.717 | 1 | <.001 | 1.543 | 1.400 | 1.701 |
|  | new 2023 combining R grants into 1 group after graduation, but before promotion to associate professor or before attrition from academia |  |  | 286.043 | 2 | <.001 |  |  |  |
|  | new 2023 combining R grants into 1 group after graduation, but before promotion to associate professor or before attrition from academia(1) | -1.427 | .142 | 100.685 | 1 | <.001 | .240 | .182 | .317 |
|  | new 2023 combining R grants into 1 group after graduation, but before promotion to associate professor or before attrition from academia(2) | -.998 | .071 | 197.223 | 1 | <.001 | .369 | .321 | .424 |
|  | Department Subunit for first appointment, 9 category |  |  | 244.970 | 8 | <.001 |  |  |  |
|  | Department Subunit for first appointment, 9 category(1) | .268 | .075 | 12.844 | 1 | <.001 | 1.308 | 1.129 | 1.514 |
|  | Department Subunit for first appointment, 9 category(2) | -.196 | .045 | 18.806 | 1 | <.001 | .822 | .753 | .898 |
|  | Department Subunit for first appointment, 9 category(3) | -.131 | .065 | 4.124 | 1 | .042 | .877 | .773 | .995 |
|  | Department Subunit for first appointment, 9 category(4) | -.468 | .042 | 124.358 | 1 | <.001 | .626 | .577 | .680 |
|  | Department Subunit for first appointment, 9 category(5) | .331 | .065 | 26.025 | 1 | <.001 | 1.392 | 1.226 | 1.580 |
|  | Department Subunit for first appointment, 9 category(6) | -.358 | .086 | 17.410 | 1 | <.001 | .699 | .591 | .827 |
|  | Department Subunit for first appointment, 9 category(7) | -.040 | .041 | .946 | 1 | .331 | .961 | .887 | 1.041 |
|  | Department Subunit for first appointment, 9 category(8) | -.211 | .074 | 8.201 | 1 | .004 | .809 | .700 | .935 |
|  | recoding into ever vs never the Direct copy of FacultyRosterNew20Top40 for Yan; Top40 Status for first appointment institution(1) | -.099 | .030 | 10.756 | 1 | .001 | .906 | .854 | .961 |
|  | Constant | 1.479 | .061 | 588.378 | 1 | <.001 | 4.389 |  |  |
| a. Variable(s) entered on step 1: months to status2_WFversion outcome, gender, race ethnicity 2023 with corrected URM groups, recoded MSQ_parent_educ_r2 continuing generation college grads as a ref group, Is med school a top 40 research institution? based on 2007-2008 all federal research expenditures, Direct copy of NEW_GQ_CAREER_PREF_CAT_faculty_r_sysmis for Yan; Career preference indicated on GQ, 3category including sysmis, recoded with fewer categories Direct copy of GQ_total_debt_cat_rsysmis for Yan; total debt indicated on GQ, sysmis included, Did trainee ever complete a research year during GME?, RANK_CD new20, Direct copy of new20TENURE_CD1_rsysmis for Yan; Tenure status at first appointment, new 2023 combining R grants into 1 group after graduation, but before promotion to associate professor or before attrition from academia, Department Subunit for first appointment, 9 category, recoding into ever vs never the Direct copy of FacultyRosterNew20Top40 for Yan; Top40 Status for first appointment institution. | | | | | | | | | |
